# Arsenic Bioaccessibility in Rice and Its Application to Derive Health-Based Limits in China

**DOI:** 10.3390/foods13172741

**Published:** 2024-08-29

**Authors:** Di Zhao

**Affiliations:** College of Resources and Environmental Sciences, Nanjing Agricultural University, Nanjing 210095, China; dizhao@njau.edu.cn

**Keywords:** arsenic, rice, bioaccessibility, safety limits, health-based

## Abstract

Arsenic (As) contamination in rice is a global public health concern, particularly in Asian countries where rice is the staple food. Current health-based limits for As in rice are typically derived from total As concentrations, resulting in overly stringent values. This study aimed to determine As bioaccessibility in rice, estimate dietary intakes of inorganic As (iAs) at different consumption rates, evaluate the cancer and non-cancer risks associated with iAs exposure through rice consumption, and assess the feasibility of deriving more accurate health-based limits for As in rice after incorporating rice As bioaccessibility. Bioaccessibility of As ranged from 60.7% to 104.5% in rice samples. Estimated iAs intake varied from 0.04 to 1.40 μg/kg BW/day at rice consumption rates of 100–300 g/day. Incorporating rice As bioaccessibility resulted in lower iAs intake estimates of 0.03–1.18 μg/kg BW/day. The non-cancer and cancer risks associated with iAs exposure are concerning for populations with higher rice consumption rates and elevated rice iAs concentrations. Health-based limits for iAs in rice for different regions across China are discussed after incorporating rice As bioaccessibility. This study contributes to the development of regional or national safety limits for As in rice, based on As bioaccessibility in rice.

## 1. Introduction

Arsenic (As) exists in the environment due to natural processes as well as human activities. Natural sources of As include volcanic emissions and the weathering of rocks. Human activities such as mining, smelting, coal burning, and the use of As-based pesticides and fertilizers can also release As into the environment [1]. Moreover, As can leach into groundwater from contaminated surface water or natural deposits, posing potential health risks when consumed by humans [2].

Humans are exposed to As via dietary and non-dietary ingestion, inhalation, and dermal absorption, with dietary intake being recognized as the primary source of As exposure in regions where As levels in drinking water are low [3,4]. Rice, which is the staple food for more than half of the global population, accumulates approximately 10 times higher As than other staple foods, making rice consumption an important contributor to dietary As intake [5,6].

A number of studies have reported the presence of As in rice, with As concentrations ranging from 0.01 mg/kg to 1.83 mg/kg [7,8]. Liu et al. [7] collected 208 rice samples from fields in South China and found that As concentrations in brown rice varied from 0.08 mg/kg to 0.67 mg/kg, with a mean value of 0.27 mg/kg. Majumder et al. [8] conducted a meta-analysis on rice grain As accumulation reported in field and market-based surveys across different countries. They observed that As concentrations in rice were highly variable, not only across the producing regions globally, but also within specific regions. Notably, rice samples from Asian countries had higher As concentrations (0.01–1.83 mg/kg) compared with those from the USA and Europe (0.03–1.16 mg/kg). These findings highlight the urgent need for continuous monitoring of As contamination in rice and its associated human health risks.

However, the health risks of As exposure depend not only on total As concentration in rice; As speciation is another important factor as As toxicity varies between different As species. It has been reported that rice from different locations differs in As speciation due to differences in rice cultivars, water management, and contamination levels [9,10]. For example, inorganic As (iAs) species, including arsenate (AsV) and arsenite (AsIII), are the dominant As species in rice from China, accounting for 57–96% of total As [11,12,13], whereas organic As species such as dimethylarsinic (DMA) are the main species found in rice from the USA [9]. Therefore, As exposure through rice consumption is a critical global public health concern, especially for the Chinese population, since inorganic As species are considered to be more toxic compared with organic As [14,15,16].

Despite numerous studies having assessed the contamination status of As in rice based on the current safety limits and quantified the associated health risks of As exposure through rice consumption [17,18,19], current health-based limits for As in rice are typically derived from total As concentrations. Few safety limits have considered bioaccessibility of As in rice, resulting in overly stringent values, since only the proportion of As that enters into the systemic circulation is bioaccessible [20,21]. Therefore, various in vitro methods have been developed to simulate human gastrointestinal conditions by measuring the fraction of As that is dissolved in simulated gastrointestinal fluids [22,23]. Among these methods, the physiologically based extraction test (PBET) method has been commonly utilized to determine As bioaccessibility in rice. However, the bioaccessibility of As varies considerably among different rice samples [22,24], posing challenges in applying bioaccessibility results from specific samples to different rice samples.

Therefore, in this study, the aims were to (1) determine the concentration and bioaccessibility of As in rice samples; (2) assess dietary iAs intakes at different consumption rates; (3) evaluate both cancer and non-cancer risks associated with iAs exposure through rice consumption; and (4) derive health-based limits for iAs in rice after incorporating rice As bioaccessibility. This study aims to support the establishment of regional or national health-based limits for As in rice with consideration of rice As bioaccessibility.

## 2. Materials and Methods

### 2.1. Rice Sample Collection and Pretreatment

A total of 117 polished, locally produced rice samples were randomly collected from two locations in China. One location was a typical As-polluted region with a long history of metal mining and smelting, while the other location was far from those mining areas. After collection, rice samples were dried, milled into fine powders, and sealed in polyethylene bags before further pretreatment.

### 2.2. Arsenic Concentrations in Rice

Rice powders were digested using the microwave-assisted method as previously described by Ma et al. [25]. Briefly, 0.3 g rice powder was digested with 5 mL of high-purity concentrated HNO_3_ (Sinopharm Chemical Reagent Co., Ltd., Shanghai, China). After cooling down to room temperature, the digest was diluted with 10 mL of 2% HNO_3_ and As concentrations were determined using inductively coupled plasma mass spectrometry (ICP-MS, NexION 300×, Perkin Elmer, Shelton, CT, USA). The ICP-MS system was calibrated before analysis using five-point calibration curves, achieving an R^2^ value greater than 0.999. For quality assurance and quality control, indium (20 μg/L) was added online to blanks, calibration standards, and digest solutions as an internal standard to correct for matrix effects and signal drift. The recoveries of internal standards were within 75–125%. For each batch of 30 samples, a reagent blank, 2 duplicate samples, and a certified reference material of NIST 1568b rice flour (National Institute of Standards and Technology, Gaithersburg, MD, USA) were included during the measurement. Repeated analysis of NIST 1568b rice flour produced As concentrations of 0.280 ± 0.013 mg/kg, which agreed well with the certified value of 0.285 ± 0.014 mg/kg. Data for total As concentrations in rice were converted to inorganic As concentrations using the ratio coefficients described by Meharg et al. [9].

### 2.3. Arsenic Bioaccessibility in Rice via PBET Method

The bioaccessibility of As in 33 rice samples with relatively high As concentrations (As: 0.11–0.39 mg/kg) was assessed using the PBET method with minor modifications [20]. Briefly, 0.2 g rice was extracted with 20 mL gastric fluid containing 1.25 g/L pepsin (TCI, Shanghai, China), 0.50 g/L citrate (Sinopharm Chemical Reagent Co., Ltd., Shanghai, China), 0.50 g/L malate (Sinopharm Chemical Reagent Co., Ltd., Shanghai, China), 420 μL lactic acid (Aladdin, Shanghai, China), and 500 μL acetic acid (Aladdin, Shanghai, China) in 1 L of Milli-Q water. The pH of the gastric solution was adjusted to 2.5 by addition of diluted HCl (Sinopharm Chemical Reagent Co., Ltd., Shanghai, China), and the solution was shaken at 37 °C for 1 h. Following gastric phase extraction, solutions were centrifuged at 4000 rpm for 10 min, with the supernatant being collected, filtered, and diluted with 2% HNO_3_ prior to ICP-MS analysis.

After the gastric phase, the extraction solution was added with 1.75 g/L bile (TCI, Shanghai, China) and 0.5 g/L pancreatin (TCI, Shanghai, China) at pH of 7.0. The solutions were then shaken for an additional 4 h. Subsequently, gastric and intestinal solutions were centrifuged at 4000 rpm for 10 min, and the supernatant was collected and filtered through a 0.45 μm membrane filter.

The extracts were stored at 4 °C until analysis by ICP-MS. The bioaccessibility of As in rice was determined by dividing the extractable As in the gastric phase or intestinal phase by the total As in the rice.

### 2.4. Health Risk Assessment and Derivation of Health-Based Limits for iAs in Rice

To assess inorganic As exposure through rice consumption, the estimated daily intake (EDI) of iAs was calculated according to Equation (1) [26]:

EDI = (C × IR × BC × EF × ED)/(BW × AT)(1)

where EDI represents the estimated daily intake of iAs, measured in micrograms per kilogram of body weight per day (μg/kg BW/day); C is the concentration of iAs in rice (mg/kg); IR is the daily ingestion rate of rice (g/day); BC is the bioaccessibility of As in rice (%), a mean value of 84.5% was used; EF is exposure frequency (day/year); ED is exposure duration (year); BW is bodyweight, the standard value of 60 kg for adults was used; AT is averaging time (day).

For the non-cancer risk, a hazard quotient (HQ) was estimated by comparing the EDI with the reference dose (RfD) using Equation (2) [26]:

HQ = EDI/RfD(2)

where RfD is 3.00 × 10^−4^ mg/kg/day for iAs. It has been concluded that there is no adverse health effect when HQ value < 1, and potential non-cancer risk is likely to occur when HQ > 1.

Regarding the carcinogenic effects, the incremental lifetime cancer risk (ILCR) was estimated as the incremental probability of an individual developing cancer over a lifetime’s exposure to iAs via rice consumption, using Equation (3) [26]:

ILCR = EDI × SF(3)

where SF is the cancer slope factor of iAs (per mg/kg/day). Generally, an ILCR value > 10^−4^ means the cancer risk is unacceptable, an ILCR value < 10^−6^ means the cancer risk is negligible.

Thus, the health-based threshold of iAs in rice for non-cancer and cancer risks can be derived based on an HQ of 1 and ILCR of 10^−4^, considering the contribution of rice to overall dietary iAs intake and rice consumption rate for populations in different regions across China. 

### 2.5. Statistical Analysis

Arsenic concentrations and bioaccessibility in rice samples are presented as means and standard deviations (SD) of three replicates. All graphs were constructed using SigmaPlot (version 14.0). Student’s *t*-test was conducted to assess significant differences in rice As concentrations between the two locations, using IBM SPSS Statistics 25 (SPSS Inc., Armonk, NY, USA) at α = 0.05.

## 3. Results

### 3.1. Total and Inorganic Arsenic Concentrations in Rice

**Figure 1:**
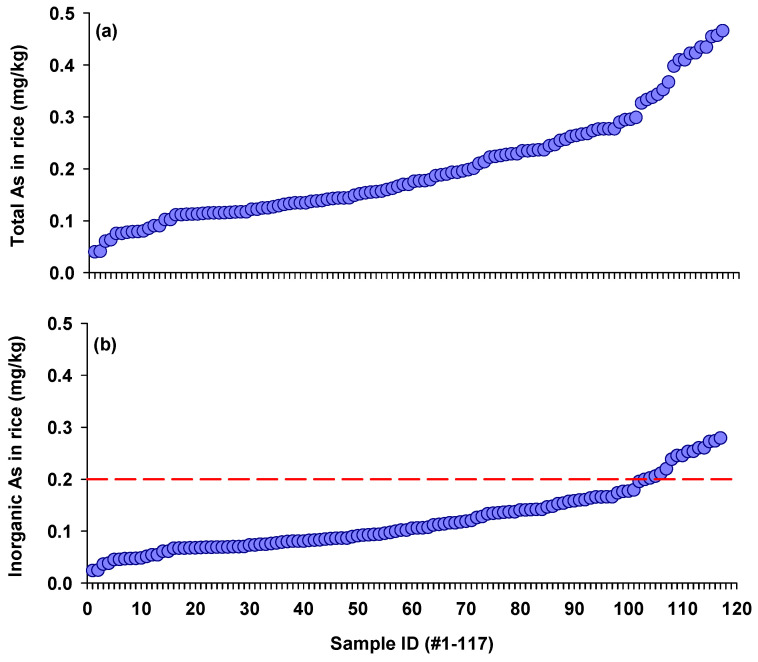
Total arsenic (**a**) and inorganic arsenic (**b**) concentrations in 117 polished rice samples collected from two regions in China. The red dashed line indicates China’s safety limit for iAs in rice (0.2 mg/kg).

A total of 117 rice samples were collected from two locations in China. The samples were numerically labeled based on increasing As concentrations (Figure 1a). The As concentrations in these rice samples were 0.04–0.47 mg/kg, with a mean concentration of 0.20 mg/kg. Notably, rice samples collected from the As-polluted region with a long history of metal mining and smelting showed significantly higher As concentration compared with rice samples from the location far from those mining activities (0.31 vs. 0.15 mg/kg). Similarly, Chen et al. [24] reported that total As concentrations in 108 rice samples from 13 major rice-producing regions in China ranged from 0.03 mg/kg to 0.33 mg/kg. Notably, rice samples with relatively higher As concentrations were collected from a major rice producing area with prolific metal industries [24]. In another study conducted in mining-impacted areas, total As concentrations in 44 rice samples ranged from 0.10 to 0.56 mg/kg [27]. In contrast, lower As concentrations of 0.01–0.23 mg/kg were found in 113 rice samples from areas far from mining and smelting activities across 19 Chinese provinces [28]. These findings suggest that the elevated total As concentrations observed in this study may be attributed to possible soil contamination from nearby mining activities.

Moreover, our findings revealed large variability in As levels in rice, consistent with observations from previous studies [7,8,13]. These variations in rice As concentrations can be attributed to several factors, including differences in soil composition, rice cultivars, and water management practices. To reduce As accumulations in rice samples, a range of mitigation methods, from agronomic measures to plant breeding and genetic modification should be employed.

Inorganic As concentrations in rice ranged from 0.02 mg/kg to 0.28 mg/kg, averaging 0.12 mg/kg (Figure 1b). The iAs concentrations observed in this study were lower than those reported in a global survey spanning 29 distinct sampling regions across six continents, where iAs concentrations were up to 0.4 mg/kg in 1180 rice samples [29]. In a large survey (*n* = 435) conducted in China, a total of 435 rice samples were collected from both markets and fields in mining-impacted areas, with the highest iAs concentration being up to 0.38 mg/kg [13]. In Bangladesh, the average iAs concentration in 965 rice samples from different districts varied considerably, ranging from 0.01 mg/kg to 0.50 mg/kg [30].

The ratio of iAs to total As in rice grains is known to vary geographically. Meharg et al. [9] found a significant correlation between iAs and total As concentrations in rice, with the ratios of iAs to total As varying across different countries. For example, the ratio was higher for rice from India and Bangladesh (0.80 and 0.72), followed by China and Italy (0.60 and 0.51), but notably lower for the USA (0.28). Similarly, Carey et al. [29] determined As speciation in rice across 29 representative rice-growing regions globally, finding relatively low levels of iAs in rice from East Africa and the Southern Indonesian islands, while rice from South America contained relatively high levels of iAs. Differences in the ratios of iAs to total As in rice may be caused by genetic variations, water management regimes, or soil properties. Since chronic exposure to low-dose iAs has been proved to be associated with various health effects, including diabetes, cardiovascular disease, and cancers [30,31,32], our findings underscore the urgent need for more vigorous measures to reduce iAs exposure via rice consumption.

Among these rice samples, approximately 12% exceeded China’s safety limit for iAs (0.2 mg/kg), all from areas with As pollution due to mining and smelting activities. Although China’s safety limit of 0.2 mg/kg is the same as has been set by the European Union and the World Health Organization for adults, the European Union has set a more stringent limit of 0.1 mg/kg for rice-based products for young infants, due to rising health concerns. Therefore, the iAs concentrations observed in these rice samples are particularly concerning for vulnerable populations, as 52.1% of the rice would not meet that infant limit.

### 3.2. Arsenic Bioaccessibility in Rice Samples

**Figure 2:**
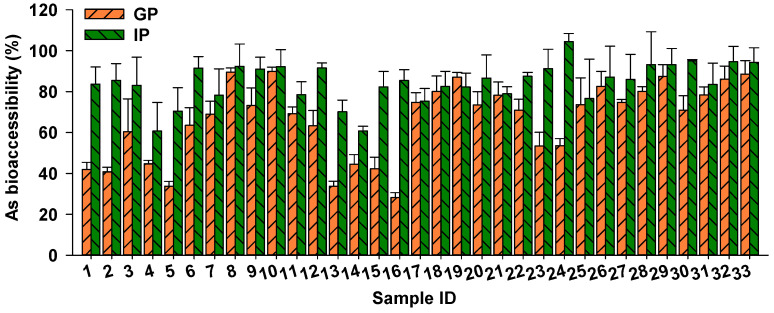
Arsenic bioaccessibility in 33 rice samples in the gastric (GP) and intestinal phases (IP), according to the physiologically based extraction test (PBET) method.

After determining the concentrations of As in rice samples, As bioaccessibility was assessed in 33 rice samples with relatively high As concentrations (As: 0.11–0.39 mg/kg), using the in vitro PBET method. The bioaccessibility of As in the gastric phase ranged from 28.2% to 89.8%, with a mean value of 65.8%. In the intestinal phase, As bioaccessibility in rice was 60.7–104.5%, with the mean value being 84.2% (Figure 2). Similar findings were reported by Chen et al. [24], with As bioaccessibility in 108 rice samples ranging from 20.1% to 82.2% (mean: 52.3%) and 47.2% to 113% (mean: 81.2%) in the gastric and intestinal phase, respectively. He et al. [33] determined As bioaccessibility in rice collected from the USA using an in vitro gastrointestinal fluid system and found similar As bioaccessibility of 53–102%.

Variation in rice As bioaccessibility may be attributed to differences in As speciation. For example, Li et al. [34] identified a strong and positive correlation between bioaccessible As and iAs (R^2^ = 0.41–0.53) in rice, while a weaker relationship was observed between bioaccessible As and organic As (R^2^ = 0.03–0.11). Similarly, a stronger correlation between bioaccessible As and iAs (R^2^ = 0.67) was observed compared with organic As (R^2^ = 0.26) in 17 rice samples collected from major rice-producing regions [24]. This suggests that iAs in rice is more bioaccessible in gastrointestinal fluids compared with organic As. In vivo studies using a swine model confirmed that 90% and 85% of administered As(III) and As(V) was absorbed from the gastrointestinal tract, respectively, whereas organic As exhibited poor absorption (20.2–31.2%), leading to the higher bioaccessibility of iAs compared with organic As [35].

Furthermore, variation in rice As bioaccessibility can be attributed to different rice varieties. Islam et al. [36] demonstrated considerable variation in As bioavailability among 12 rice genotypes, ranging from 24.7% to 94.1%. Among these rice samples, Binadhan-8 showed the highest As bioavailability of 94.1%, followed by Binadhan-5 at 92.8%, while Binadhan-10 showed the lowest As bioavailability at 24.7%. Additionally, the Japonica variety exhibited lower As bioaccessibility compared with the Indica variety, probably due to the higher proportion of iAs in Indica rice [24]. Therefore, prioritizing the selection of cultivars with low As accumulation and bioaccessibility is crucial for minimizing As exposure through rice consumption.

### 3.3. Estimated Daily Intake of Inorganic Arsenic

The health risks associated with iAs exposure through rice consumption depend not only on the concentration of iAs in the rice, but also the rate of rice consumption. Therefore, the daily intake of iAs was estimated using Equation (1) for three different consumption rates, including 100 g/day, 200 g/day, and 300 g/day.

**Figure 3:**
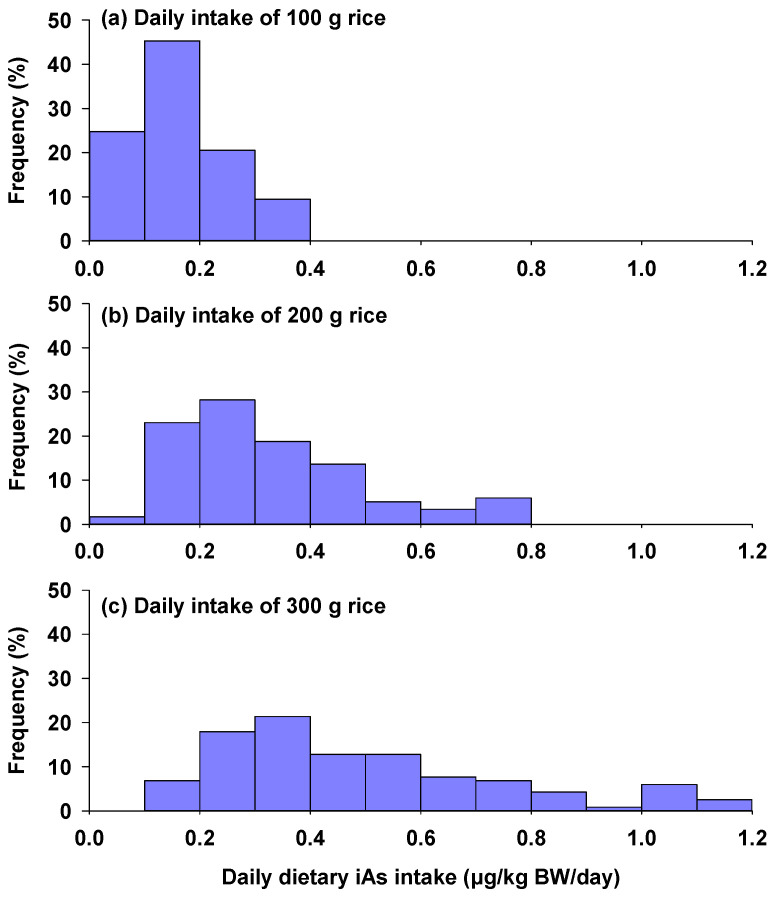
Frequency distribution of dietary inorganic arsenic intakes calculated for different rates of rice consumption ((**a**) daily intake of 100 g rice; (**b**) daily intake of 200 g rice; (**c**) daily intake of 300 g rice) after incorporating rice As bioaccessibility. A bodyweight of 60 kg for adults was used in the calculation.

For populations consuming 100 g of rice daily, estimated daily intake of iAs ranged from 0.03 to 0.39 μg/kg BW/day (Figure 3a), with the highest proportion of dietary iAs intake falling between 0.1 and 0.2 μg/kg BW/day. For those consuming 200 g of rice daily, the iAs intake was estimated to be 0.07–0.79 μg/kg BW/day, with the highest proportion of intake falling in the range of 0.2–0.3 μg/kg BW/day (Figure 3b). At a daily rice consumption rate of 300 g, estimated daily intake of iAs ranged from 0.10 to 1.18 μg/kg BW/day (Figure 3c), well below the benchmark As dose for 0.5% increased incidence of lung cancer (BMDL_0.5_) set at 3 μg/kg BW/day by the WHO. Similarly, Li et al. [18] observed substantial regional variation in dietary iAs intake across different regions in China, attributed to diverse dietary habits. Specifically, populations in the North had the lowest iAs intake of 0.47 μg/kg BW/day, whereas those in the South had the highest iAs intake of 0.88 μg/kg BW/day. Additionally, both rural and urban populations exhibited comparable daily iAs intake levels, averaging 0.71 μg/kg BW/day. In another study conducted in China with a focus on urban populations, the average daily iAs exposure through rice intake was estimated to be 0.18 μg/kg BW/day, with a 95th percentile interval from 0.001 to 1.22 μg/kg BW/day [36]. Similar levels of dietary iAs intake have been observed in populations from other Asian countries, where rice is the staple food for local populations. For example, Mondal et al. [37] reported that the lifetime daily intakes of iAs from rice consumption in three regions of India were 0.3–0.84 μg/kg BW/day.

Conversely, for populations with lower rice consumption, such as the general population in the UK, average iAs intake was notably lower, ranging from 0.024 to 0.25 μg/kg BW/day [38]. For the Italian population, the mean chronic dietary iAs exposure ranged from 0.07 to 0.19 μg/kg BW/day, while the 95th percentile exposure ranged from 0.23 to 0.78 μg/kg BW/day, based on the 2012–2014 Total Diet Study [39]. Notably, dietary iAs intake varies among different age groups, with the highest mean dietary iAs intake being observed in infants and toddlers (0.19 μg/kg BW/day), followed by children (0.15 μg/kg BW/day), adolescents (0.09 μg/kg BW/day), adults (0.07 μg/kg BW/day), and the elderly (0.067 μg/kg BW/day). These findings underscore the importance of monitoring and managing dietary iAs to protect public health, particularly among younger populations who are more susceptible to As-related risks.

### 3.4. Health Risk Assessment of iAs in Rice

**Figure 4:**
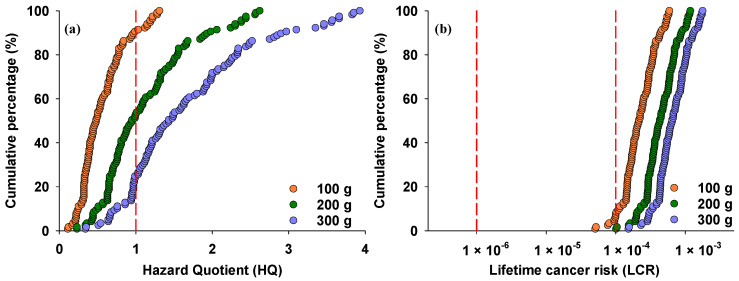
Cumulative percentage of hazard quotient (**a**) and lifetime cancer risk (**b**) due to rice consumption, calculated for different rates of rice consumption. Red dashed lines indicate threshold values of hazard quotient and lifetime cancer risk associated with iAs.

The potential health risks associated with iAs exposure through rice consumption were estimated using Equations (2) and (3) (Figure 4). According to Environmental Protection Agency (EPA) guidelines, adverse health effects may occur when HQ ≥ 1, while HQ <1 means no adverse health effect. With a daily rice intake of 100 g, HQ values ranged from 0.11 to 1.31, with 9.4% exceeding the threshold (Figure 4a). The data indicate that iAs concentrations in rice exceeding 0.21 mg/kg may pose non-cancer risks at this consumption rate. When daily rice intake increased to 200 g and 300 g, HQ values increased to 0.23–2.62 and 0.34–3.93, respectively, with 47% and 75% exceeding the threshold value of 1. These data suggest that rice containing more than 0.11 mg/kg and 0.07 mg/kg of iAs may pose non-cancer risks at daily consumption rates of 200 g and 300 g, respectively.

For ILCR, the EPA recommends an acceptable range of 1 × 10^−6^–1 × 10^−4^. At a daily rice intake of 100 g, the individual ILCR from rice iAs intake ranged from 5.07 × 10^−5^ to 5.90 × 10^−4^, with 93% exceeding the threshold value of 10^−4^ (Figure 4b). When daily rice intake increased to 200 g and 300 g, all ILCR values for iAs exceeded 10^−4^, indicating high risk from exposure to iAs in rice due to the high daily ingestion rate among local populations.

### 3.5. Deriving Health-Based Limits for iAs in Rice

Health-based limits for iAs in rice were assessed for populations across China with consideration of As bioaccessibility in rice (Table 1). Overall, the contribution of rice to overall dietary iAs intake ranged from 41.7% to 64.1%, with a national mean value of 57.8%. Additionally, rice ingestion rates vary for populations across China, ranging from 123.8 g/day in North China to 326.6 g/day in South China, with a national mean value of 238.3 g/day [18]. Considering the contribution of rice to overall dietary iAs intake and rice ingestion rates for populations in different regions, adults would face potential non-cancer risk if the safety limits of 0.04–0.07 mg/kg were reached. However, the current safety limit for iAs in rice (0.2 mg/kg) in China exceeds these threshold values in all regions, indicating that the current safety limit is not sufficient to protect human health in all populations across China with different ingestion rates, even after considering As bioaccessibility. Regarding cancer risks, adults would face potential cancer risk if the safety limit of 0.01 mg/kg were reached.

## 4. Conclusions

Elevated As concentrations were observed in rice samples, especially from mining impacted regions. Incorporating bioaccessibility leads to more accurate health risk estimation. Our assessment indicates that the current limit for iAs in rice may be inadequate to safeguard human health since it may result in HQ and ILCR values higher than the acceptable risk level even after considering rice As bioaccessibility. Given the high consumption rates of rice in China, particularly in Southern regions, there is an urgent need for more stringent limits to protect populations in China.

Current limits for iAs in rice rarely consider rice As bioaccessibility. This study is a preliminary exploration of deriving health-based limits for As in rice in China, with consideration of rice As bioaccessibility. However, the variability in As bioaccessibility among different rice samples challenges the accuracy of current risk assessments based on average values. Developing reliable predictive models for As bioaccessibility in rice through comprehensive large-scale assessments is crucial for improving the accuracy of risk assessments. Moreover, dietary habits vary greatly among populations, and the reliance on average rice consumption rates does not account for individual variability in dietary habits. Looser limits may be insufficient to protect vulnerable populations or those in contaminated areas, while stricter limits may overprotect human health. Further studies should integrate individual dietary variations to better inform health-based limits. Additionally, regulation limits for iAs in rice vary across countries and organizations. As a result, iAs concentrations in rice that meet the limits in some countries may exceed the limits set by others, leading to restrictions on the global rice trade.

Overall, this work contributes to the development of regional or national safety limits for As in rice by considering As bioaccessibility. The findings will help to derive more accurate health-based limits for As in rice across regions in China.

## Figures and Tables

**Table 1:** Health-based limits for iAs in rice for populations living in different regions of China.

Region	Contribution of Rice to Overall Dietary iAs Intake (%)	Ingestion Rate (g/day)	Limits for iAs in Rice with Consideration of Bioaccessibility (mg/kg)		GB 2762-2022 [40]
			Based on Cancer Risk	Based on Non-Cancer Risk	
National	57.8	238.3	0.01	0.05	0.2
Urban	52.4	217.8	0.01	0.05	
Rural	59.9	246.2	0.01	0.05	
North	41.7	123.82	0.02	0.07	
South	64.1	326.65	0.01	0.04	
Coastal	52.6	235.09	0.01	0.05	
Inland	59.6	244.63	0.01	0.05	

## Data Availability

The original contributions presented in the study are included in the article, further inquiries can be directed to the corresponding author.
